# *Neisseria gonorrhoeae* antimicrobial resistance patterns and associated risk factors in women of childbearing potential in northwestern Ethiopia

**DOI:** 10.1186/s12905-024-02898-3

**Published:** 2024-01-31

**Authors:** Engdawork Demissie, Azanaw Amare, Muluken Birhanu, Mucheye Gizachew

**Affiliations:** 1 Department of Medical Laboratory Sciences, School of Medicine and Health Sciences, Jigjiga University, Jijiga, Ethiopia; 2 Department of Medical Microbiology, College of Medicine and Health Sciences, University of Gondar, Gondar, Ethiopia; 3 Department of Medical Microbiology, Immunology and Parasitology, Addis Ababa University, and Assosa University, Addis Ababa, Ethiopia

**Keywords:** *Neisseria gonorrhoeae*, Antimicrobial resistance, Women of reproductive age

## Abstract

**Backgrounds:**

*Neisseria gonorrhoeae* causes gonorrhea and poses public health problems, including antimicrobial resistance. Current data on gonorrhea in prenatal participants in the study area are required. Thus, we aimed to identify gonorrhea prevalence, antimicrobial resistance, and risk factors among antenatal care clinic visitors in northwestern Ethiopia.

**Methods:**

A cross-sectional study was conducted from March to August 2022 at the University of Gondar Comprehensive Specialized Hospital. We recruited 278 study participants using convenient sampling techniques. Sociodemographic, clinical and behavioral risk factors were recorded using pre-tested questionnaires. Endocervical swabs were collected by a physician, transported to the microbiology laboratory, immediately inoculated into modified Thayer-Martin medium, and it was incubated at 37 °C for 24–48 hours. Gram staining and biochemical tests were used to identify the organism. AMR testing was performed using disc diffusion and E-test methods. Data were entered in EPI-info version 7 and exported and analyzed in SPSS version 26. A *p*-value ≤0.05 was considered as statistically significant. Results were presented in words, tables and figure.

**Results:**

Of 278 subjects enrolled, majority (44.6%) were 26–35 years, with a mean age of 29.9 (SD = ±7.2) years, 69.4% were urban residents, and 70.5% were married. Twenty-one (7.6%) participants had gonorrhea. Overall antimicrobial resistance ranged from 19 to 100%. High resistant to tetracycline (100%) and penicillin (85.7%) were observed by both tests. Ciprofloxacin resistance was 52.4% by disc diffusion and 85.7% by E-test. By E-test, all isolates were sensitive to ceftriaxone, cefixime, azithromycin and spectinomycin; however, 7 (33.3%), 9 (42.9%), 9 (42.9%) and 5 (23.8%) isolates showed resistant to these antibiotics with disk method. Prevalence of beta-lactamase producing *Neisseria gonorrhoeae* was 85.7%. Alcohol consumption (*p = 0.032*), condom-free sexual practice (*p = 0.010*), multiple sexual partners (*p < 0.001*), pelvic pain (*p = 0.018*), and dysuria (*p = 0.021*) revealed increased risk of infection.

**Conclusions:**

Compared with many previous studies in Ethiopia, we found high prevalence, antimicrobial resistance, and beta-lactamase-positive isolates. Multiple sexual partners, alcohol consumption, not using condom, pelvic pain and dysuria were predictors of this infection. Continuous large-scale monitoring of pathogen is essential for its prevention and control.

## Background

Gonorrhea, caused by *N.gonorrhoeae*, is the second most common sexually transmitted bacterial infections remaining a serious public health concern worldwide [1]. This obligate human host-adapted pathogen was described for the first time by Albert Neisser in Gram-stained microscopy of urethral discharge in 1879 [2]. *N.gonorrhoeae* is a diplococcus, Gram-negative microorganism that belongs to the bacterial class Betaproteobacteria and the family Neisseriaceae, which comprises the genus Neisseria and other genera such as Kingella and Eikenella [3].

Globally, the prevalence and incidence of curable sexually transmitted infections (STIs) remains high, with approximately over one million new infections per day [4, 5]. As of 2016, 376 million people were newly infected with curable STDs worldwide, 86.9 million were infected with gonorrhea, and the global prevalence among adults aged 15–49 was 0.9% [6]. According to the World Health Organization’s (WHO) annual report, gonorrhea is one of the most common STIs worldwide, with 87 million new infections. About 4 million of these are found in Europe, North America, Australia and New Zealand. More than 80 million gonococcal infections occur in low- and middle-income countries such as Asia, Africa, Latin America and the Caribbean [7].

WHO estimates that sub-Saharan Africa is responsible for about 40% of the global sexually transmitted diseases (STD) burden [8]. Symptomatic and asymptomatic STIs are a major cause of morbidity in developing countries and can lead to infertility, cervical cancer, pregnancy complications [9], and pelvic inflammatory disease (PID) [10]. Additionally, there is evidence that STDs may increase the risk of contracting and transmitting the human immunodeficiency virus (HIV) [11]. Several studies in sub-Saharan Africa have found that women of childbearing age are at increased risk of contracting STIs. Social behavioral risk factors for STIs in this group included having low educational level, unmarried, experiencing multiple sexual partners, high alcohol consumption and drug use, and early sexual debut [12]. In addition, commercial sex workers are at increased risk of contracting STIs [13].

Approximately 35% of Ethiopia’s population is young between 15 and 24 years of age, and is highly susceptible to STDs [14]. STI surveillance conducted in eight health facilities in different parts of Ethiopia found that younger people were more susceptible to STIs [15].

*Neisseria gonorrhoeae* has been acquired AMR to all antimicrobials recommended earlier as first-line or second-line empirical treatment of gonorrhea (for example, sulfonamides, penicillins, tetracyclines, fluoroquinolones, early generation macrolides / erythromycin, and cephalosporins / cefuroxime) [16]. Antimicrobial resistance varies greatly from country to country. Data on prevalence, antimicrobial resistance patterns, and associated factors over consecutive years are therefore of great importance, especially for *Neisseria gonorrhoeae,* a highly AMR bacterium [17].

Ethiopia’s health policy follows WHO recommendations, advocates symptomatic treatment of STIs, and encourages routine surveillance and screening for syphilis and HIV in pregnant women. Treatable STDs remain a neglected topic in research despite their well-known prevalence worldwide [18]. The prevalence of gonococcal disease among women of childbearing age is largely neglected in Ethiopia due to the lack of a national gonococcal testing programme. Data are often incomplete and unreliable. This is because the data generated from diagnosing this syndrome are not specific enough for the diagnosis of gonorrhea, and there is no adequate reporting mechanism [19]. Therefore, the aim of this study was to investigate the prevalence of gonococci, their AMR patterns, and associated risk factors in symptomatic women of childbearing potential attending the outpatient department (OPD) of the University of Gondar Comprehensive Specialized Hospital (UoG-CSH).

## Materials and methods

### Study design, period, and area

A cross-sectional study was conducted from March to August 2022 at the Gynecology OPD, UoG-CSH located in Gondar town. The city is located in the Amhara National Region Province in northwestern Ethiopia, 750 km from Addis Ababa, the capital of Ethiopia. In 2021, Gondar had a population of 378,331 [20]. The hospital provides various health services, including gynecology, and obstetrics for the community. In addition, the hospital has accredited laboratories and more than 1200 beds, providing referral services for more than five million patients from the surrounding areas and/or regions.

### Source population

The source population was all patients who visited the UoG-CSH gynecology outpatient clinic during the study period.

### Study population

The study population consisted of sexually transmitted infections suspected women of childbearing potential who met eligibility criteria during the study period.

### Inclusion and exclusion criteria

All women of childbearing age with signs or symptoms suggestive of a sexually transmitted infections participated in the study. Those who have taken antibiotics within the past 2 weeks, who were menstruating, pregnant women and patients with serious illness or mental disorders who failed to provide information were excluded from the study.

### Dependent variables

Prevalence of *N. gonorrhoeae* and antimicrobial resistance.

### Independent variables

Socio-demographic factors (age, education, place of residence, occupation. Marital status), clinical factors (vaginal discharge, pain urination, lower abdominal pain, history of STIs, history of abortion, diagnosed for infertility, non-prescribed antimicrobial usage, antimicrobial use in the last three months, completion of prescribed antimicrobials), and behavioral factors (alcohol consumption, chewing khat, cigarette and shisha smoking, number of sexual partners, use of condom).

### Sample size and sampling techniques

The sample size was determined by using a single-population proportion statistical formula.

$$\boldsymbol{n}=\frac{\left(\boldsymbol{Z}\boldsymbol{\alpha } \boldsymbol{l}\textbf{2}\right)\textbf{2}\boldsymbol{p}\left(\textbf{1}-\boldsymbol{p}\right)}{\left(\boldsymbol{d}\right)\textbf{2}}$$

Where; n = minimum sample size required for the study.

Zα/2 = 1.96, (confidence interval).

p = expected prevalence (prevalence of *N****.*** *gonorrhoeae* = 20.8%, this prevalence is taken from the previous study conductesd in Gondar [21].

d = tolerable error between the sample and true population, which is 5% (0.05).

q = 1- *p* = 1–0.208 = 0.792

$$\boldsymbol{n}=\frac{\left(\boldsymbol{Z}\boldsymbol{\alpha } \boldsymbol{l}\textbf{2}\right)\textbf{2}\boldsymbol{p}\left(\textbf{1}-\boldsymbol{p}\right)}{\left(\boldsymbol{d}\right)\textbf{2}}=\frac{\left(\textbf{1}.\textbf{96}\right)\textbf{2}\ast \textbf{0.208}\left(\textbf{0.792}\right)}{\left(\textbf{0.05}\right)\textbf{2}}=\textbf{253.139}\approx 253$$

Finally, considering the 10% non-response rate, the final sample size was 253 + (0.1 × 253) = 278.

All consecutive STIs suspected childbearing potential who participated in UoG-CSH during the study period were included in the study by convenient sampling technique.

### Data collection and laboratory methods

#### Questionnaire

All study variables were considered using a predesigned three-part questionnaire. The first part contains socio-demographic information, the second part contains information on medical history, and the third part contains information on factors related to sexual behavior and substance use. The questionnaire was initially prepared in English, translated into Amharic (the local language) and retranslated into English by linguists for analysis and reporting. For those who were illiterate, we employed interviewing technique. To protect their privacy or to maintain the confidentiality issues, we conducted interviews with the illiterate people in a separate room.

#### Sample collection and handling

Endocervical swabs were collected aseptically by a physician at the gynecology outpatient clinic. Samples were then shipped by using Amie’s transport medium (to maintain viability of pathogens) to the UoG-CSH microbiology laboratory and immediately inoculated into appropriate media [22].

#### Laboratory methods

##### Isolation of *Neisseria gonorrhoeae*

Swab specimens taken from suspected patients were inoculated into modified Thayer-Martin medium (MTM) (Oxoid, Basingstoke and Hampshire, UK). The inoculated plates were then incubated at 37 °C in a humidified atmosphere supplemented with 5% CO2 using a candle jar. *N. gonorrhoeae* produces small, raised, gray, glossy colonies on MTM after overnight incubation [22]. Isolates were identified as *N. gonorrhoeae* by colony morphology and Gram staining reaction and confirmed by biochemical examination [23].

##### Identification of *Neisseria gonorrhoeae*

Biochemical testing was performed on pure colonies, and *N. gonorrhoeae* was identified based on biochemical reactions. A carbohydrate utilization test, an oxidase test, and a catalase test were included in the biochemical tests to identify the species. *N. gonorrhoeae* was distinguished from other Neisseria, Moraxella and Kingellas genera by acid production from glucose. Therefore, we performed a carbohydrate utilization study using the analytical profile index to identify *N.gonorrhoeae* and Haemophilus kit strips (API NH) (Oxoid, Basingstoke and Hampshire, UK,). *N. gonorrhoeae* are oxidase and catalase positive and ferment glucose, but not maltose, sucrose or lactose [23].

#### Antimicrobial susceptibility test

Antimicrobial susceptibility testing of the *N. gonorrhoeae* isolates was performed by using the Clinical Laboratory Standards Institute (CLSI) Kirby-Bauer disc diffusion, and E-test methods [24]. Three to five pure colonies were transferred to sterile saline tubes to generate a bacterial suspension comparable to a 0.5 McFarland standard. A sterile cotton swab was used to spread the bacteria evenly across the surface of the chocolate agar containing 1% Vitox supplement. Isolates were tested against the following antimicrobials: Penicillin (P 10 IU), Tetracycline (TE 30 μg), Ciprofloxacin (CIP 5 μg), Ceftriaxone (CRO 30 μg), Cefixime (CFM 5 μg), Cefoxitin (FOX 30 μg) Spectinomycin (SPT 100 μg), Amikacin (AMK 15 μg);, Clindamycin (CLN 2 μg), Gentamycin (GEN 15 μg),and Azithromycin (AZT 15 μg); All were from Oxoid, Basingstoke, Hampshire, England. Zone diameters were measured using a calibrated ruler and interpreted as susceptible, intermediate, and resistant based on CLSI guideline [24]. Isolates resistant to one of the currently recommended treatments (ceftriaxone or azithromycin) and at least two other antibiotics were classified as multidrug-resistant against *Neisseria gonorrhoeae* (MDR-NG) using the MIC technique [25]. The E-test was carriedout by using Etest strips as specified by the manufacturer (AB Biodisk, Stockholm, Sweden) against ceftriaxone, cefixime, penicillin G, spectinomycin, ciprofloxacin, azithromycin and tetracycline to confirm or validate isolates resistant to antibiotics that were found using the disk diffusion method. *N. gonorrhoeae* isolates were also tested for β-lactamase production by the chromogenic cephalosporin method using nitrocefin freeze-dried powder (Oxoid, Hampshire, UK) [26].

#### Data management and quality control

##### Data quality control

A preliminary test was conducted to confirm the validity of the questionnaire. Data collectors received half-day training in data collection procedures and interview techniques. All media were prepared following standard procedures for media preparation at the Medical Microbiology Laboratory, UoG-CSH, according to manufacturer’s instructions. All materials, equipment and procedures were controlled during the pre-analytical, analytical and post-analytical stages of quality assurance. The sterility of freshly prepared media was checked by incubating 5% of the batch overnight at 35–37 °C prior to use. Performance testing was performed by inoculating a known control strain, *N.gonorrhoeae* ATCC 49226, as recommended by CLSI for quality control [24]. Reference strains were obtained from the Ethiopian Public Health Institute.

#### Statistical analysis

Data were checked for completeness, coded and entered by using EPI-info version 7, and exported to the SPSS version 26 for analysis. A frequency analysis was performed to determine the frequencies of the independent variables compared to the frequencies of the dependent variables. Bivariate and multivariate logistic regression analyzes were performed to determine factors associated with *N. gonorrhoeae* prevalence. A variable with *p-value ≤ 0.25* in bivariate logistic regression was tested for statistically significant association in multivariable analysis. Raw and adjusted odds ratios were calculated to quantify the strength of associations between outcome variables and risk factors. An independent variable with a *p-value < 0.05* in multivariable analysis was considered statistically significant.

## Results

### Socio-demographical characteristics of the study participants

A total of 278 women of childbearing age (15–49 years) with suspected sexually transmitted infections were participated in the study. Majority of the study participants were 124 (44.6%) between ages of 26 and 35 years, with a mean age of 29.9 (SD = ± 7.2) years age. Of the study participants, 193 (69.4%), were urban dwellers and 196 (70.5%) were married (Table 1).

**Table 1:** Sociodemographic characteristics of the study participants (*n* = 278) in northwestern Ethiopia, March to August 2022

Variables	Frequency N (%)	*Neisseria gonorrhoeae* infection	
		Positive n (%)	Negative n (%)
Age group			
15–25	85(34.6)	7(8.2)	78(91.8)
26–35	124 (44.6)	9(7.3)	115(92.7)
36+	69 (24.8)	5(7.2)	64(92.8)
Educational status			
Illiterate	28 (10.1)	2(7.1)	26(92.9)
Elementary	79 (28.4)	7(8.9)	72(91.1)
High school	121 (43.5)	11(9.1)	110(90.9)
College/university	50 (18.0)	1(2)	49(98)
Residence			
Urban	194 (69.8)	12(6.2)	182(93.8)
Rural	84 (30.2)	9(10.7)	75(89.3)
Occupation			
Farmer	17 (6.10)	3(17.6)	14(82.4)
Housewife	60 (21.6)	4(6.7)	56(93.3)
Government worker	63 (22.7)	4(6.3)	59(93.7)
Commercial Sex workers	3 (1.1)	0	3(100)
Merchant	116 (41.7)	8(6.9)	108(93.1)
Student	19 (6.8)	2(10.5)	17(89.5)
Marital status			
Single	55 (19.8)	3(5.5)	52(94.5)
Married	196 (70.5)	14(7.1)	182(92.9)
Divorced	17 (6.1)	3(17.6)	14(82.4)
Widowed	10 (3.6)	1(10)	9(90)
**Total**	**278(100%)**	**21(7.6%)**	**257(92.4%)**

### Clinical history and syndromic characteristics of participants

Of all patients included in the study, 233 (83.8%) experienced vaginal discharge, 112 (40.3%) felt abdominal pain, 111 (39.9%) had painful urination, 44 (15.8%) %) had a history of sexually transmitted infections, 31 (11.2%) had a history of abortion and six (2.2%) were diagnosed with infertility (Table 2).

**Table 2:** Clinical history and syndromic characteristics of the study participants (*n* = 278) in northwestern Ethiopia, March to August 2022

Variables	Frequency N (%)	*Neisseria gonorrhoeae* infection	
		Positive n (%)	Negative n (%)
Vaginal discharge			
Yes	233 (83.8)	20 (8.6)	213 (91.4)
No	45 (16.2)	1 (2.2)	44 (97.8)
Pain during urination			
Yes	111 (39.9)	14 (1.3)	97 (98.7)
No	167 (60.1)	7 (4.2)	160 (95.8)
Lower abdominal pain			
Yes	112 (40.3)	14 (12.5)	98 (87.5)
No	166 (59.7)	7 (4.2)	159 (95.8)
History of STIs			
Yes	44 (15.8)	8 (18.2)	36 (81.8)
No	234 (84.2)	13 (5.5)	121 (94.5)
History of abortion			
Yes	31 (11.2)	4 (13)	27 (87)
No	247 (88.8)	17 (6.9)	230 (93.1)
Diagnosed for sterility			
Yes	6 (2.2)	2 (33.3)	4 (66.7)
No	272 (97.8)	19 (7)	253 (93)
Non-prescribed antibiotic usage			
Yes	180 (64.7)	13 (7.2)	167 (92.8)
No	98 (35.3)	8(8.1)	90 (92.9)
Antibiotics usage in the last three months			
Yes	139 (50.0)	17 (12.2)	122 (87.8)
No	139 (50.0)	4 (2.9)	135 (97.1)
Completion of prescribed drugs			
Yes	133 (47.8)	15 (12.3)	118 (87.7)
No	145 (52.2)	6 (4.1)	139 (95.9)
**Total**	**278(100%)**	**21(7.6%)**	**257(92.4%)**

### Substance usage and sexual risk behavior of participants

Of the 278 participants, 70 (25.2%) were regularl drinking alcohol, 13 (4.7%) were chewing khat (*Catha edulis*), one (0.4%) was smoking cigarette, six (2.2%) were smoking Shisha, 35 (13.6%) had multiple sexual partners in the past 3 months, and 203 (73%) did not use condom (Table 3).

**Table 3:** Substance use and sexual risk behaviors of the study participants (*n* = 278) in northwestern Ethiopia; March to August 2022

Variables	Frequency N (%)	*Neisseria gonorrhoeae* infection	
		Positive n (%)	Negative n (%)
Alcohol drinking			
Yes	70 (25.2)	13 (18.5)	57 (81.5)
No	208 (74.8)	8 (3.9)	200 (96.1)
Chewing khat			
Yes	13 (4.7)	5 (38.5)	8 (61.5)
No	167 (60.1)	7 (4.2)	160 (95.8)
Cigarette smoking			
Yes	1 (0.4)	1 (100)	0
No	277 (99.6)	20 (7.2)	257 (92.8)
Shisha smoking			
Yes	6 (2.2)	3 (50)	3 (50)
No	272 (97.8)	18 (6.6)	254 (93.4)
Sexual partner number			
One	243 (87.4)	9 (3.7)	234 (96.3)
More than one	35 (13.6)	12 (34.3)	23 (65.7)
Do you use a condom			
Yes	75 (27)	2 (2.7)	73 (97.3)
No	203 (73)	19 (9.4)	184 (90.6)
**Total**	**278(100%)**	**21(7.6%)**	**257(92.4%)**

### Prevalence of *Neisseria gonorrhoeae*

The overall prevalence of *N.gonorrhoeae* among reproductive-age women was 21(7.6%)(95%CI: 4.7–11.3) (Tables 1, 2 & 3). The frequencies are almost the same across the different age groups (Table 1). The prevalence of infection was higher among rural residents (10.7 vs.6.2% urban), the married person (7.1 vs. 5.5% single), farmers (17.6% vs. 6.9% merchants, and 6.3% government workers) (Table 1).

### Antimicrobial resistance pattern of *Neisseria gonorrhoeae*

A total of 11 antimicrobials were tested to identify AMR patterns in *N.gonorrhoeae*. The overall AMR rates for the isolates in this study ranged from 19 to 100%. High rates of resistance were observed for tetracycline (100%), penicillin (85.7%) by both disk diffusion and E-test methods, and 52.4% for ciprofloxacin by disk diffusion and 85.7% by E-test method. Isolates showed 66.6 and 61.9% susceptibility to ceftriaxone and spectinomycin, respectively, using the disc diffusion method. However, all isolates were susceptible to ceftriaxone, cefixime, azithromycin and spectinomycin by E-test. Nineteen (85.7%) isolates were beta-lactamase producers. Regular alcohol consumption (*p* = 0.032), condomless sex (*p* = 0.010), and multiple sex partners (*p* < 0.001) were associated with increased risk of infection (Table 4).

**Table 4:** AMR patterns of *N. gonorrhoeae* isolated from the study participants in northwestern Ethiopia; March to August 2022

Antimicrobial Agent	Antimicrobial resistance pattern of *Neisseria gonorrheae* (*n* = 21)					
	Disc Diffusion result			E-test (MIC) result		
	S n(%)	I n (%)	R n(%)	S n (%)	I n (%)	R n (%)
Ceftriaxone	14 (66.7)	–	7(33.3)	21 (100)	0	0
Cefixime	12 (57.1)	–	9 (42.9)	21 (100)	0	0
Azithromycin	12 (57.1)	–	9 (42.9)	21 (100)	0	0
Ciprofloxacin	6(28.6)	4(19)	11(52.4)	3(14.3)	–	18(85.7)
Spectinomycine	13 (61.9)	3 (14.3)	5 (23.8)	21 (100)	0	0
Tetracycline	0	–	21(100)	0	0	21 (100)
Penicillin	0	3 (14.3)	18 (85.7)	3(14.3)	0	18(85.7)
Cefoxitine	13 (61.9)	2 (9.5)	6 (28.6)	–	–	–
Gentamycin	5 (23.8)	10 (47.6)	6 (28.6)	–	–	–
Amikacine	13 (61.9)	3 (14.3)	5 (23.8)	–	–	–
Clindamycin	12 (57.1)	5 (23.8)	4 (19)	–	–	–

Key: *AMR* antimicrobial resistance, *MIC* minimum inhibitory concentration test, *S* susceptible, *I* intermediate, *R* resistance, *n* number

### The prevalence of beta-lactamase producing *N. gonorrhoeae*

**Fig. 1:**
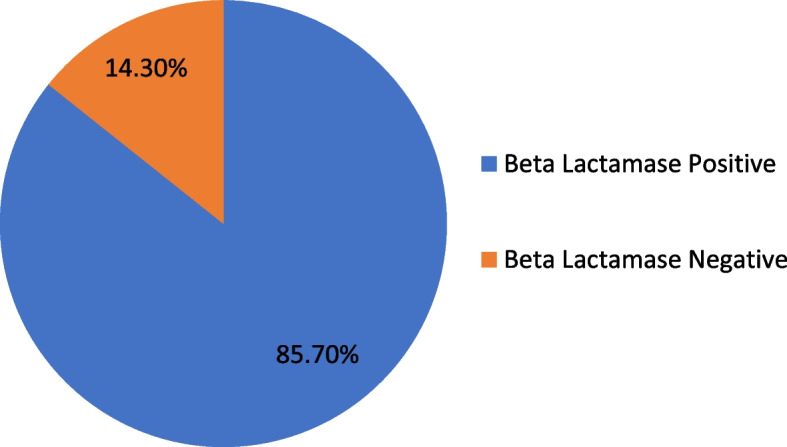
Proportion of Beta Lactamase producing *Neisseria gonorrhoeae* in northwestern Ethiopia; March to August 2022

In this study, 19 out of all *N. gonorrhoeae* isolates (85.7%) were confirmed to be positive for beta-lactamase by both disc diffusion and E-testing methods (Fig. 1).

### Factors associated with *Neisseria gonorrhoeae* infection

Both bivariate and multivariate logistic regression analyzes were performed to examine the effects of independent variables on gonococcal infection. Participants who regularly consumed alcohol had a four-fold higher rate of gonococcal infection (*p* = 0.032, AOR = 4.2, (95% CI: 1.13–15.65) compared with those who did not drink alcohol. Participants who did not use condoms had a 14-fold higher risk of developing gonorrhea (*p* = 0.010, AOR = 13.93, (95% CI: 1.89, 102.59) than those using condoms. Participants with two or more sexual partners were 25 times more likely to develop gonorrhea (*p* < 0.001, AOR = 24.9, 95% CI: 5.30, 117.00) compared with those who had one sexual partner. Study participants complained of lower abdominal pain (*p* = 0.018, AOR = 4.7 (95% CI: 1.30, 17.13) and those who experienced painful micturition (*p* = 0.021, AOR = 4.7 (95% CI: 1.26, 17.57) had almost four times the risk of contracting *N. gonorrheae* infection as compared to their counterparts (Table 5).

**Table 5:** Logistic regression analyses for factors associated with *N. gonorrhoeae* infections among study participants in northwestern Ethiopia; March to August 2022

Variables	Category	*Neisseria gonorrhoeae*		COR(95%CI)	*P-value*	AOR(95%CI)	*p-value*
		+ve	-ve				
Alcohol drinking	Yes	13	57	5.708(2.253,14.431)	< 0.001	4.2(1.13–15.65)	0.032*
	No	8	200			1	
Chewing chat	Yes	5	8	9.727(2.853,33.156)	< 0.001	0.03(0.28,3.29)	0.326
	No	16	249			1	
Shisha smoking	Yes	3	3	14.111(2.656,74.974	< 0.001	0.15(0.006,4.207)	0.268
	No					1	
Number of sexual partners	> 1	12	23	13.565(5.170,35.590)	< 0.001	24.9(5.30, 117.00)	< 0.00*
	1	9	243			1	
Using condom	NO	19	184	3.769(0.856,16.591)	0.079	13.93(1.89,102.59)	0.010*
	Yes	2	73			1	
Vaginal discharge	Yes	20	213	4.131(0.540,31.597)	0.0172	9.28(0.53,162.86)	0.127
	No	1	44			1	
Lower abdominal pain	Yes	14	98	3.245(1.266,8.320)	0.014	4.7 (1.30, 17.13)	0.018*
	No	7	159			1	
Pain during urination	Yes	14	97	3.299(1.286,8.480)	0.013	4.7(1.26, 17.57)	0.021*
	No	7	160			1	
History of STI	Yes	8	36	3.778(1.46,9.754)	0.006	2.18(.45, 10.55)	0.329
	No	13	221			1	
History of discourage/abortion	Yes	4	27	2.004(0.68,6.9393)	0.240	3.27(0.38, 27.81)	0.277
	No	17	230			1	
Diagnosed for sterility	Yes	2	4	6.658(1.145,38.728)	0.035	0.441(0.65, 15.76)	0.653
	No	19	253			1	

Abbreviations: *COR* Crude Odds Ratio, *AOR* Adjusted Odds Ratio, *CI* Confidence Interval, *+ve* Positive, *−ve* Negative

## Discussion

WHO includes *N. gonorrhoeae* on list of top infectious diseases worldwide for development of new interventions [27, 28]. It is also reported to be the most prevalent STD in low- and middle-income countries [7]. Although many studies have been conducted on gonococcal prevalence and AMR patterns in Ethiopia, few current data are available for women of childbearing potential [29].

In this study, the prevalence of *N. gonorrhoeae* among female participants of reproductive age with suspected STI was 7.6% (95% CI: 4.7, 11.3). This is comparable to studies conducted in Mekelle, Ethiopia (10.04%) [30], in Italy (7.4%) [31]; Iran (7.2%) [32]; South Africa (6.8%) [33]; Swaziland (6.0%) [12]; Jimma, Ethiopia (9.4%) [29], and Gambella, Ethiopia (5.0%) [34]. However, it was higher than studies reported from Ethiopia: Hawassa (3.3%) [35]; Bahir dar (4.7%) [36]; and Hawassa again (4.3%) [37]; (1.6%) in Kenya [38]; (1.8%) in Gambia [39]; (1.9%) in South Africa [40]; and 1.0% in Brazil [41]. On the other hand, our result was lower than the studies from Addis Ababa, Ethiopia (11.4%) [42]; and Ghana (28.3%) [43]. These prevalence variations may be due to differences in target population, sample size, study design, and laboratory capacity. This relatively high culture confirmed prevalence of *N. gonorrhoeae* in the present study may also be due to a lack of differential diagnosis, which may mean that many patients remain untested and untreated. Syndromic treatment of cases may accelerates disease spread and may also lead to increased AMR [44].

Regarding risk factor assessment, the present study identified alcohol consumption, multiple sexual partners, condom use failure, lower abdominal pain and dysuria as risk factors for infection. The results showed that gonorrhea was significantly associated with alcohol consumption (p = 0.032), as shown in other studies conducted in different parts of Ethiopia such as in Gambella [34]; Jimma [29];a nd Bahir Dar [36]; and in Kenya [38]. This may be because alcohol is primarily used as a social lubricant. This can lead to serious consequences such as unprotected or non-consensual sex, as inhibitions are broken when drinking and women may make the wrong decisions [45]. In addition, multiple sexual partners *(p < 0.001)* and no condom use *(p = 0.010)* were significantly associated with gonococcal disease. Italy [31]; Brazil [41]; Bulgaria [46]; Kenya [43]; and Ethiopia [29] also reports that multiple sexual partners pose a risk of gonococcal infection. Further studies were conducted in Hawassa [47]; Bahir Dar [36]; and Jimma [29] from Ethiopia also found that not using a condom could be a risk factor for gonorrhea. This may be because in patriarchal societies like Ethiopia, the subordination of women seems legitimate. They were afraid to negotiate safe sex because they could not negotiate condom use or refuse forced or non-consensual sex [48], and thus, it may lead to gonococcal infection. Furthermore, in this study, patients complaining of pelvic pain (p = 0.018) and dysuria (*p = 0.021*) had an increased risk of gonococcal infection, which is consistent with results reported in Hawassa, Ethiopia [37]; and Bulgaria [46]. A history of lower abdominal (pelvic) pain and / or difficulty of urinating (dysuria) may indicate a STI.

Understanding AMR patterns is fundamental to the correct treatment of infectious diseases, including gonorrhea. Regional gonorrhea AMR surveillance programs have been established in developed countries [49]. However, developing countries have a high disease burden and rapidly increasing AMR. Although national protocols recommend ceftriaxone as first-line treatment for gonorrhea, 33.4% of isolates were resistant to ceftriaxone using disc diffusion method, which is higher than the rate of resistance reported in different parts of Ethiopia: Addis Ababa [42]; Jimma (0%) [29]; and Gambella (0%) [34]; 0% in Philippines [50]; and 28% inVietnam [51]. But, we did not find resistant isolates by the E-test method, indicating that ceftriaxone remains the drug of choice for the treatment of gonorrhea. The rate of ceftriaxone resistance using disc diffusion is lower in this study than in Ghana (85.5%) [43]. Extended-spectrum cephalosporins are the only first-line antibiotics recommended for the empirical treatment of simple gonorrhea in some countries, but many strains of *N. gonorrhoeae* with reduced susceptibility to ceftriaxone are found worldwide. Excessive and/or irrational use of the drug without bacteriological diagnosis may be the cause of this alarming emergence of resistance. This can cause serious problems for gonorrhea, which quickly becomes unmanageable with these antimicrobials [52].

In the present study, 52.4% isolates were ciprofloxacin resistant using disc diffusion method which is comparable with a report from Vietnam (54%) [51]. However, it was higher than the studies reported from Ethiopia(12.9%) [29]; Ghana (46.2%) [43]; and China (3%) [53].

This may be due to the widespread use of ciprofloxacin for treating other bacterial infections, and STIs using a symptomatic approach. This is evidenced by the fact that the current national treatment guidelines for STIs in Ethiopia recommend the use of ciprofloxacin for the treatment of groin and genital ulcers [54]. On the other hand, we found lower results than the studies from Addis Ababa, Ethiopia (75%) [42]; and the Philippines (78%) [50].

High level of penicillin resistance (85.7%) was observed using both disc diffusion and E-test methods in this study, comparable to other studies in Zambia (85.2%) [55]; and (86%) in South Africa [56]. However, it is higher than Ethiopian reports such as: 80.6% in Jimma [29]; and 68.5% in Addis Ababa [42]; and 33% in South Africa [33].

Using both disc diffusion and E-test methods, we found 100% resistant to tetracycline. This is consistent with 100% resistant isolates reported in Ethiopia [34]; and South Africa, and 94% in Ghana [43]. However, this result is higher than other reports such as 54.8% [29]; (69.6%) [30], and 71.87% [42] in Ethiopia; and 68.9% in Zambia [55]. This may be due to the emergence of beta-lactamase-producing bacteria that are penicillin-resistant.

Penicillin or tetracycline resistance caused by plasmid- or chromosomal-mediated mechanisms has been found to be common in South Africa [56]. Furthermore, gonorrhea and its etiology, AMR, are known to vary greatly between countries and regions. This is likely due to differences in treatment protocols and methods of diagnosing and treating the disease in different countries [57].

The prevalence of gonococcal infections and the alarming increase in resistance to most antimicrobials in current use are due to both the indiscriminate and intensive use of antimicrobials and the STIs syndrome management system. In addition, genetic variation within organisms and acquisition of resistance genes from commensal species may contribute to increased AMR in *N. gonorrhoeae*. Numerous studies have shown the importance of gene exchange in the emergence of AMR in pathogenic *Neisseria* species [58, 59]. We also found that 19 (85.7%) of the isolates produced beta-lactamase, which is consistent with 83.8% results from Thailand [60].

## Conclusions and recommendations

The study found that the rate of gonorrhea infection was relatively high compared to results from other parts of the country. According to our findings, we observed high levels of resistance to tetracyclines, penicillins and ciprofloxacin. Majority of the isolates were betalactamase producers. Pelvic pain, dysuria, alcohol consumption, multiple sexual partners, and condom use failure were most important risk factors for gonorrhea. Large scale surveillance for *N. gonorrhoeae* and its AMR is warranted for appropriate management of patients with gonococcal infection. Agar dilution or E-test reference testing is also needed for accurate assessment of resistance. Even though penicillin is no longer recommended for gonorrhea treatment, beta-lactamase production by *N. gonorrhoeae* should be monitored because of the potential for the development of extended-spectrum beta-lactamases.

## Data Availability

No datasets were generated or analysed during the current study.

## Abbreviations

AMR: Antimicrobial Resistance
AST: Antimicrobial Susceptibility Test
CLSI: Clinical and Laboratory Standards Institute
HIV: Human Immunodeficiency Virus
MDR-NG: Multi-Drug Resistance *Neisseria gonorrhoeae*
STIs: Sexually Transmitted Infections
UoG-CSH: University of Gondar Comprehensive Specialized Hospital.
